# Hybrid Physics-Guided Neural Network for Vibration Sensor Nonlinearity Correction

**DOI:** 10.3390/s26196165

**Published:** 2026-09-29

**Authors:** Alexander P. Lyapin, Faizulddin Ebrahimi, Evgeny D. Agafonov, Viktor S. Ratushnyak, Julia Schnitzer

**Affiliations:** 1 Department of Computational and Information Technology, Siberian Federal University, Krasnoyarsk 660041, Russia; aplyapin@sfu-kras.ru; 2 Department of Economics, Shenzhen MSU-BIT University, Shenzhen 518712, China; 3 Department of Fuel Supply and Lubricants, Siberian Federal University, Krasnoyarsk 660041, Russia; eagafonov@sfu-kras.ru; 4 Department of System Analysis and Operations Research, Reshetnev Siberian State University of Science and Technology, Krasnoyarsk 660037, Russia; 5 Department of Train Traffic Management Systems, Institute of Railway Transport, Krasnoyarsk 660028, Russia; ratushnyak_vs@krsk.irgups.ru; 6 Department of Computer Science and Media, Brandenburg University of Applied Sciences, 14770 Brandenburg an der Havel, Germany; julia.schnitzer@th-brandenburg.de

**Keywords:** deep learning, physics-guided neural network, MEMS accelerometer, vibration sensor, nonlinear distortion, hybrid model, residual learning, low-pass filter, sensor error correction

## Abstract

Accelerometers built on micro-electromechanical systems (MEMS) play a critical role in structural monitoring and machinery diagnostics; however, their accuracy suffers from intrinsic nonlinearities—dead zones, hysteresis, saturation, and colored noise. Conventional physics-based correction methods are interpretable yet cannot capture complicated hysteretic behavior, while purely neural-network approaches generalize poorly and lack a physical foundation. This paper proposes a hybrid architecture that combines a residual convolutional neural network with a physics-guided low-pass filter prior, fused through an attention-gated mechanism. The CNN learns only the residual nonlinearity; the filter supplies a steady, band-limited baseline. We validate the model on two simulated scenarios—a noise-dominant track and a nonlinear-dominant track—across three random seeds. The resulting Hybrid LPF-CNN outperforms a standalone CNN by 15.2% and an LSTM by 33.5% on the severely nonlinear track, reaching a mean R^2^ of 0.9970 and an RMSE of 0.0179 g. On the noise-dominant track, it reaches R^2^ = 0.9407 and RMSE = 0.0800 g, surpassing both CNN and LSTM baselines. The model is also stable across seeds (σ=0.0001 in R^2^) and gives a legible breakdown of the correction it applies. Our systematic architectural search revealed that a dual-encoder design with attention-gated fusion—where raw and filtered signals are processed separately and combined via a learnable spatial gate—provides the optimal balance between stability, accuracy, and interpretability. Even basic physics priors substantially improve the performance, stability, and interpretability of deep learning models for sensor error correction.

## 1. Introduction

A sensor detects a physical input—heat, light, motion, pressure—and converts it into a measurable electrical signal. A vibration sensor, or accelerometer, is a transducer that specifically gauges the acceleration, velocity, or displacement of a mechanical oscillation. These sensors matter for condition monitoring, fault diagnosis, and dynamic response estimation: they pick up the temporal and frequency signature of dynamic motion, which provides information about the operational state and structural integrity of an engineered system. They show up across a wide range of fields. In structural health monitoring (SHM), they enable early detection of damage in bridges, buildings, and aerospace structures [1,2,3]. In machinery diagnostics, they facilitate predictive maintenance of rotating equipment such as turbines, motors, and pumps [4,5,6]. In inertial navigation, they provide critical motion data for autonomous vehicles, drones, and handheld devices [7,8].

However, MEMS accelerometers—despite their low cost, small size, and low power consumption—suffer from significant nonlinearities that degrade measurement fidelity. These include dead zones near zero displacement due to stiction and mechanical friction [9], rate-independent hysteresis that introduces memory-dependent errors and phase distortion [10], soft saturation caused by the finite output voltage range of the readout electronics that clips high-amplitude signals, and colored noise (1/f flicker noise) that dominates at low frequencies and corrupts critical low-frequency vibration components [11,12].

Conventional physics-based correction methods are interpretable yet cannot capture complicated hysteretic behavior, while purely neural-network approaches generalize poorly and lack a physical foundation. Physics-based techniques—including the Extended Kalman Filter (EKF) [13], autoregressive models with exogenous inputs (ARX), and classical low-pass filters—provide interpretable corrections grounded in physical principles. The EKF, for instance, can model dead-zone and saturation behavior using a nonlinear measurement function, while the ARX model captures linear dynamics through a finite impulse response structure. However, these methods struggle with hysteretic memory effects because they lack internal state variables to represent history-dependent behavior. The low-pass filter, though effective at reducing high-frequency noise, cannot restore signal components lost due to saturation or correct phase distortion caused by hysteresis. Consequently, purely analytical approaches are insufficient for high-precision correction when significant nonlinearities are present [14,15,16].

Data-driven methods using deep learning—particularly Multilayer Perceptrons (MLPs), Convolutional Neural Networks (CNNs), and Long Short-Term Memory (LSTM) networks—have shown remarkable capability to learn complex nonlinear mappings directly from data [17]. These models can capture hysteresis, dead-zone effects, and saturation without explicit mathematical formulations, making them well-suited for sensor error correction where the underlying physical mechanisms are difficult to model analytically [18]. However, purely data-driven approaches have important limitations. First, they require large amounts of labeled training data, which can be expensive or impractical to obtain for real sensors. Second, they generalize poorly outside their training distribution, often memorizing the inverse mapping for specific distortion parameters rather than learning a transferable correction. Third, they operate as black boxes, providing little insight into the physical basis of their predictions, which undermines trust in safety-critical applications [19,20].

Recent advances in Physics-Informed Neural Networks (PINNs) have sought to address these limitations by embedding governing physical equations into the loss function. Reference [21] demonstrated that PINNs can solve forward and inverse problems involving partial differential equations by penalizing the residual of the governing equations during training. The authors of [22] further extended the paradigm to various scientific domains, showing that physics constraints can regularize neural networks and improve generalization. In the context of structural dynamics, physics-informed approaches have been applied to condition monitoring of rotating shafts and metamodeling of nonlinear structures. Reference [23] proposed a self-supervised physics-informed network with frequency-domain priors for heterodyne interferometry error compensation.

However, several challenges remain for PINN-based sensor correction. First, embedding complex differential equations in the loss function significantly increases the computational cost and can lead to training instability, particularly when the physical model is stiff or contains discontinuities. Second, the physics residual often requires automatic differentiation of the network output, which introduces additional gradient computations and memory overhead. Third, for sensor error correction, the governing physical equations may not be known exactly—the forward sensor model may be partially unknown or contain unmodeled dynamics such as aging effects or temperature drift [24,25,26].

Hybrid approaches that combine physics-based priors with data-driven learning have emerged as a promising middle ground. Reference [27] developed a multi-sensor data fusion system for monitoring precision in thin-wall lens barrel turning. Reference [28] proposed physics-informed graph neural networks for sim-to-real structural response prediction. Reference [29] constrained neural-network predictions using mass-spring-damper models in structural dynamics. Reference [30] addressed phase deviation in semi-active suspension control through inertial suspension compensation, highlighting the critical importance of signal phase fidelity in vibration control applications.

Still, most hybrid approaches focus on embedding complex differential equations into the network architecture, leaving simpler, more interpretable linear priors—such as low-pass filters and regularized ARX models—largely unexplored within a residual-learning framework for sensor error correction. Moreover, there is no thorough comparison yet of state-of-the-art deep learning models for vibration sensor correction against physics-based baselines like Kalman filters, ARX models, and low-pass filters. Additionally, existing methods tend to get validated under narrow operating conditions, without real testing across fundamentally different distortion regimes—from noise-dominated scenarios to strongly nonlinear ones. Without that kind of benchmarking, practitioners have little to go on when picking an approach for a given application.

In order to find a reliable and efficient design, we methodically assessed a number of hybrid architectures during our inquiry. The first efforts were cascading an Extended Kalman Filter with a CNN and parallel LSTM-CNN structures; however, they either produced overcorrected outputs that enhanced high-frequency noise or failed to converge because of gradient instability. After much testing, we found that the best combination of stability, accuracy, and interpretability is provided by a dual-encoder architecture with an attention-gated fusion mechanism, in which the low-pass filter prior and the raw distorted signal are processed through different feature encoders and combined via a learnable spatial gate. This discovery forms the core architectural novelty of our work. We adopt the term “physics-guided” to distinguish our method from PINNs that embed physics in the loss function. In our framework, physics enters as a prior, not as a residual constraint. This paper addresses these gaps with a hybrid architecture for correcting nonlinear distortions in MEMS vibration sensors: a residual convolutional neural network combined with a physics-guided linear baseline. The core idea is to use a zero-phase low-pass Butterworth filter—which encodes the band-limited nature of mechanical vibrations—as a strong, physically meaningful prior, then train a 1-D CNN to learn only the residual nonlinearity the prior cannot capture. Because the CNN only has to learn a small correction term, training remains stable; the physics prior guarantees a baseline that never deteriorates; and an attention-gated fusion mechanism learns when to trust the prior versus the data-driven correction, adapting across different signal regimes. The main contributions of this research are as follows:

(1) The proposal of an evaluation framework for nonlinear MEMS vibration sensor error correction, which compares three physics-based baselines—the Extended Kalman Filter, the regularized ARX model, and the zero-phase low-pass filter—against deep learning models such as MLP, CNN, and LSTM across two distinct operating scenarios. (2) A novel hybrid architecture (Hybrid LPF-CNN) is developed that fuses a residual CNN with a physics-guided low-pass filter prior through an attention-controlled mechanism, letting the model learn only the nonlinear residual while keeping a stable, interpretable baseline, with experimental results demonstrating that the hybrid model consistently outperforms both the physical prior and standalone deep models, achieving an R^2^ of 0.9970 on the strongly nonlinear track and 0.9407 on the noise-dominated track, with consistent performance across three independent random seeds. (3) A statistical analysis is conducted of distribution plots and confidence intervals, demonstrating the method’s reproducibility and potential for generalization, with all figures and code released for full replication, alongside a breakdown of the hybrid model showing how the CNN correction term builds on the physics-based prior, including what the learned residual actually captures. These contributions will serve as the foundation for future research into hardware implementation.

## 2. Materials and Methods

### 2.1. Sensor and Signal Modeling

The most prevalent nonlinear faults found in commercial products are simulated in a MEMS accelerometer. NumPy 1.26.4, SciPy 1.11.4, PyTorch 2.1.0, and Matplotlib 3.8.2 were used to implement all simulations and neural network training in Python 3.10.12. As of 19 August 2026, the software packages can be found at https://www.python.org, https://numpy.org, https://scipy.org, https://pytorch.org, and https://matplotlib.org. The Data Availability Statement contains a link to the code and data used in this investigation. The sensor is assumed to be a 16-bit device, and quantization noise is below the modeled noise floor, so it is omitted. The sampling frequency of 1000 Hz was selected to satisfy the Nyquist criterion for the excitation bandwidth. The sensor model incorporates four distinct nonlinear phenomena:

1. Dead Zone: Near zero displacement, the sensor output remains zero due to stiction and mechanical friction. This is modeled as

(1) $$h_{\mathrm{dead}}\left(x\right)=\left\{\begin{array}{cc} 0, & |x|<\delta \\ x-\mathrm{sgn}(x)\delta , & |x|\geq \delta \end{array}\right.$$

where δ is the dead-zone threshold [9].

2. Hysteresis: Rate-independent hysteresis is modeled using the Prandtl–Ishlinskii (PI) model, which captures memory-dependent behavior through a weighted superposition of elementary play operators [10]:

(2) $$h_{\mathrm{hyst}}\left(t\right)=\sum_{i=1}^{N}w_{i}\;F_{r_{i}}\left[x\right]\left(t\right)$$

where $F_{r_{i}}$ is the play operator with threshold $r_{i}$ and $w_{i}$ are weights. This model is widely used for piezoelectric and MEMS devices.

3. Soft Saturation: The finite output voltage range of the sensor’s readout electronics causes saturation, modeled by a hyperbolic tangent function [9,10,11]:

(3) $$h_{\mathrm{sat}}\left(x\right)=S\times \tanh\left(x/S\right)$$

where *S* is the saturation limit.

4. Colored and White Noise: MEMS accelerometers exhibit both white thermal noise and flicker (1/f) noise [11,12]:

(4) $$n\left(t\right)=n_{w}\left(t\right)+n_{c}\left(t\right)$$

where $n_{w}\left(t\right)∼N\left(0,\sigma_{w}^{2}\right)$ is white noise, and $n_{c}\left(t\right)$ is colored noise with power spectral density $S\left(f\right)\propto f^{-\alpha }$.

The overall sensor output combines these effects:

(5) $$y\left(t\right)=h_{\mathrm{sat}}\left(h_{\mathrm{hyst}}\left(h_{\mathrm{dead}}\left(x\left(t\right)\right)\right)\right)+n\left(t\right)$$

Signals are normalized to a peak amplitude between 0.3 g and 1.0 g, where $g=9.81\;{m/s}^{2}$ denotes the standard acceleration due to gravity. This normalization ensures that all reported RMSE and MAE values in units of g correspond to physically meaningful acceleration levels typical of structural vibration monitoring applications [1,2].

Table 1 summarizes the parameter values for Track A (noise-dominant) and Track B (nonlinear-dominant).

### 2.2. Physics-Based Baselines

Three linear/analytical techniques have been developed to act as benchmarks and as inputs to the hybrid model.

#### 2.2.1. Extended Kalman Filter (EKF)

The Extended Kalman Filter (EKF) uses a random walk state model and a nonlinear measurement function to simulate the sensor’s dead zone and saturation behavior while excluding hysteresis (since hysteresis introduces memory effects that the EKF state formulation cannot represent without augmenting the state vector). The state equation is $x_{k}=x_{k-1}+w_{k}$, with process noise covariance *Q*; the measurement equation is $y_{k}=h\left(x_{k}\right)+v_{k}$, with measurement noise covariance *R*. The function h(·) is defined as

(6) $$h\left(x\right)=\left\{\begin{array}{cc} 0, & |x|<\delta \\ S\times \tanh\;\left(\frac{x-\mathrm{sgn}(x)\delta }{S}\right), & |x|\geq \delta \end{array}\right.$$

The EKF recursively estimates the state using the linearized Jacobian $H_{k}={\left.\partial h/\partial x\right|}_{x_{k}^{-}}$. To obtain peak performance, the filter parameters *Q* and *R* are adjusted independently for each track (noise-dominant vs. nonlinear-dominant).

#### 2.2.2. Autoregressive with eXogenous Input (ARX)

To avoid instability, a simplified linear FIR (finite impulse response) model was used rather than a full ARX. The prediction for time *t* is a linear mixture of the latest *w* distorted samples:

(7) $${\hat{y}}_{\mathrm{ARX}}\left(t\right)=\sum_{j=1}^{w}\beta_{j}\;y\left(t-j\right)$$

Ridge regression is used to estimate the coefficients $\beta \in R^{w}$, preventing ill conditioning:

(8) $$\hat{\beta }=\arg\min_{\beta }\left\{∥Y_{\mathrm{clean}}-Y_{\mathrm{dist}}{\beta ∥}_{2}^{2}+\lambda {∥\beta ∥}_{2}^{2}\right\}$$

where $Y_{\mathrm{clean}}$ represents actual clean samples, $Y_{\mathrm{dist}}$ is the design matrix of previous distorted windows, and $\lambda =10^{-2}$ is the regularization value. This FIR model represents the sensor’s linear dynamics without relying on recursive predictions, which prevents error buildup.

#### 2.2.3. Low-Pass Filter (LPF)

The distorted signal is filtered using a fourth-order zero-phase Butterworth low-pass filter. This filter encodes the physical precondition that vibration signals are band-limited and that high-frequency components above a cutoff frequency $f_{c}$ are essentially noise. The transfer function is

(9) $$H\left(s\right)=\frac{1}{1+{\left(s/\omega_{c}\right)}^{2n}},\;\omega_{c}=2\pi f_{c}$$

with $f_{c}=300$ Hz and n=4. Forward–backward filtering (filtfilt) eliminates phase lag; therefore, the filtered signal $y_{\mathrm{LPF}}\left(t\right)$ is a smoothed version of the distorted input.

The filtered signal is expressed as $y_{\mathrm{LPF}}\left(t\right)=F^{-1}\left\{H\left(s\right)\cdot F\left\{y\left(t\right)\right\}\right\}$, where F is the Fourier transform. This $y_{\mathrm{LPF}}\left(t\right)$ works as the physics-guided prior that is subsequently fed as the second input channel (alongside the raw distorted signal y(t)) into the hybrid architecture described in Section 2.4.

The digital implementation is obtained via the bilinear transform, and the difference equation for a single pass is

(10) $$y_{\mathrm{LPF}}\left[n\right]=\sum_{k=0}^{M}b_{k}\;y\left[n-k\right]-\sum_{k=1}^{N}a_{k}\;y_{\mathrm{LPF}}\left[n-k\right]$$

with M=N=4. The zero-phase output is then computed by filtering in both directions.

### 2.3. Deep Learning Baselines

Three neural network topologies have been constructed, which work directly on the distorted signal to reconstruct the clean vibration.

#### 2.3.1. Multi-Layer Perceptron (MLP)

The MLP receives a sliding window of the distorted signal only:

(11) $$u\left(t\right)=y\left(t-w:t\right)\in R^{w}$$

where *w* is 20. Three hidden layers with 256, 128, and 64 neurons make up the network; batch normalization, GELU activation, and dropout (0.1) come next. The scalar prediction $\hat{x}\left(t\right)$ is the result. Crucially, no clean samples from the past or present are utilized in training or inference, guaranteeing a fair comparison with alternative approaches.

#### 2.3.2. Convolutional Neural Network (CNN)

A 1-D residual CNN with multiscale dilation serves as a solid data-driven baseline. The architecture contains the following elements:

- A stem convolution (kernel size 7 and 16 channels);
- Four residual blocks with dilation rates 1, 2, 1, and 4, each includes two convolutional layers with batch normalization and GELU;
- The final head has 8 channels and uses 1×1 convolution to produce a single-channel output;
- A learnable skip connection (initialized to zero) that combines the input and the processed signal.

The CNN receives the distorted sequence y(t) as input and produces the reconstructed sequence ${\hat{x}}_{\mathrm{CNN}}\left(t\right)$.

#### 2.3.3. Bidirectional LSTM

To capture long-range temporal dependencies, a single-layer bidirectional LSTM is used, with 64 hidden units in each direction. The LSTM processes the input sequence in both forward and backward directions, and the outputs are projected through a linear layer to produce the reconstructed sequence ${\hat{x}}_{\mathrm{LSTM}}\left(t\right)$. The LSTM converges smoothly without the oscillations that would result from stochastic optimization, confirming the training setup’s reliability.

### 2.4. Proposed Hybrid Model: Hybrid LPF-CNN

**Figure 1:**
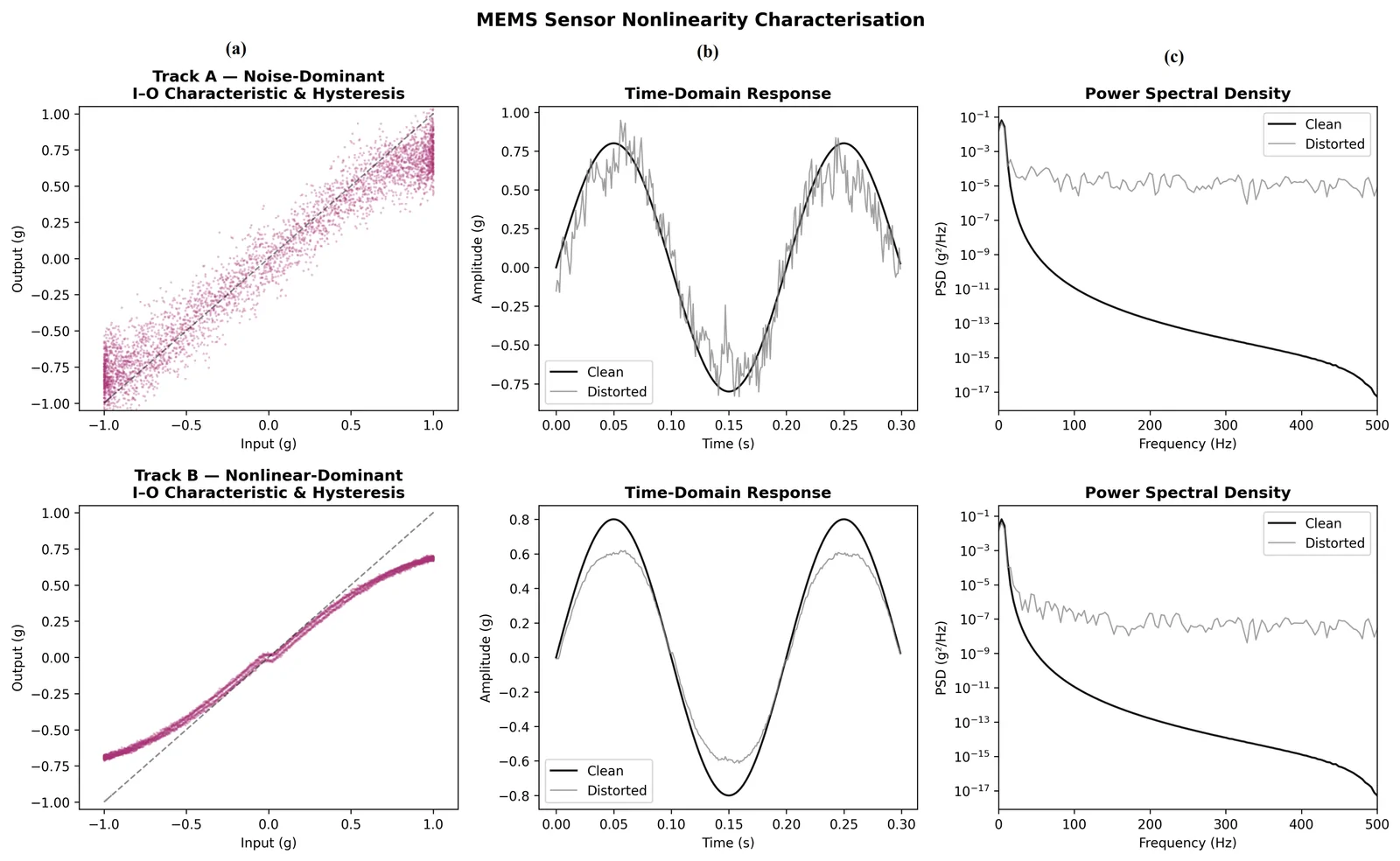
Nonlinear analysis of the MEMS sensor model: (**a**) I/O characteristics, (**b**) temporal response, and (**c**) PSD.

This study’s main novelty is a hybrid design that uses an attention-controlled refinement network and dual encoder to mix the raw distorted signal with the low-pass filtered signal (physics prior). As seen in Figure 1, the sensor shows considerable saturation and hysteresis, requiring sophisticated correction techniques. Two input channels, y(t) (distorted) and $y_{\mathrm{LPF}}\left(t\right)$ (prior), are needed for the model.

**Figure 2:**
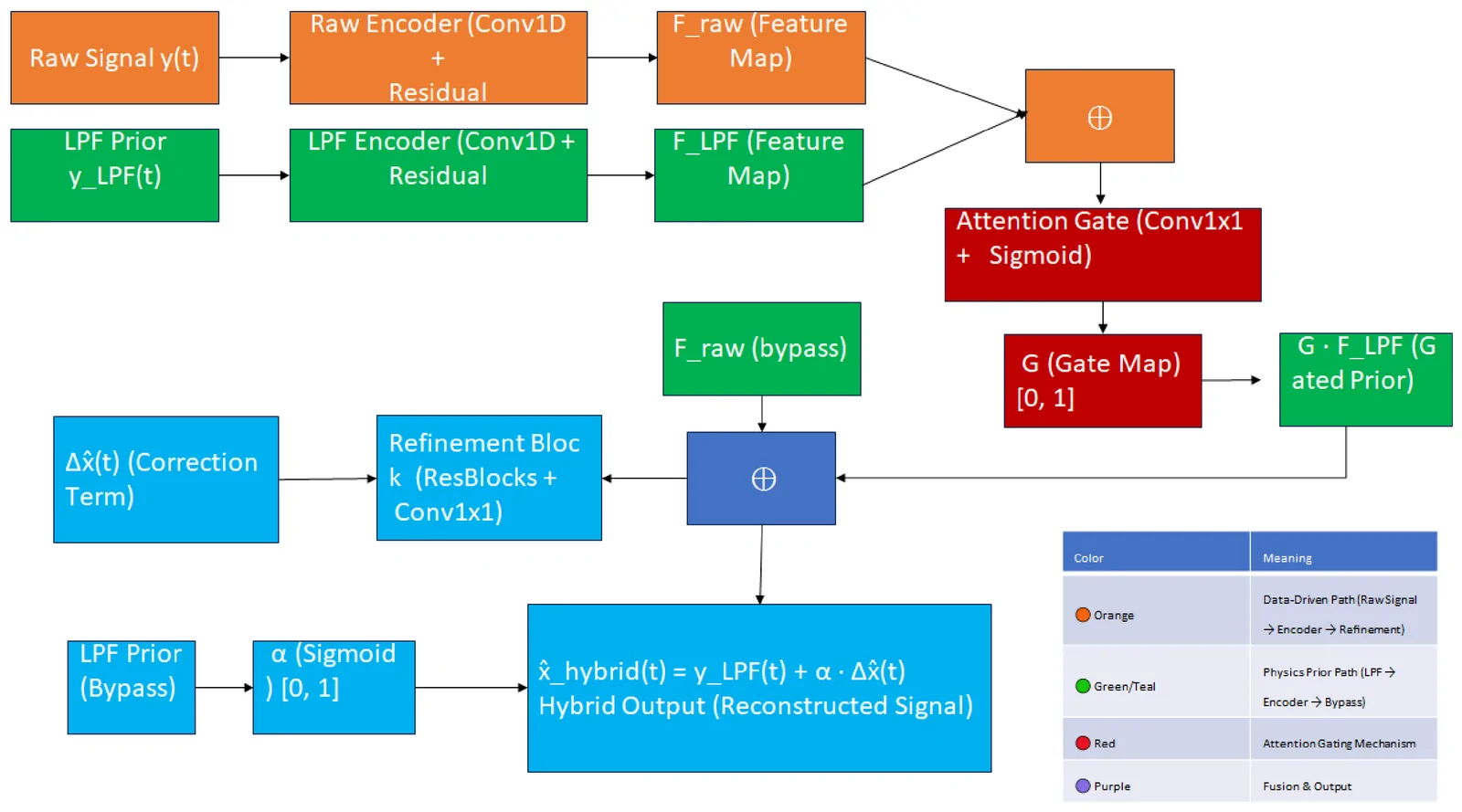
Schematic architecture of the proposed Hybrid LPF-CNN model.

The suggested Hybrid LPF-CNN architecture is shown schematically in Figure 2. Through separate feature encoders, the model receives two inputs: the LPF prior $y_{\mathrm{LPF}}\left(t\right)$ and the raw distorted signal y(t). The LPF characteristics are modulated by a spatial weight map *G* created by an attention gate. The correction term $\Delta \hat{x}\left(t\right)$ is created by concatenating the raw features $F_{\mathrm{raw}}$ with the gated prior $G⊙F_{\mathrm{LPF}}$ and passing it through a refinement block. The LPF prior and a learnable blending parameter α are combined in the final output: ${\hat{x}}_{\mathrm{hybrid}}=y_{\mathrm{LPF}}+\alpha \cdot \Delta \hat{x}\left(t\right)$.

As shown in Figure 2, the architecture consists of three primary parts.

**Feature encoders** are independent 1-D convolutional stacks (kernel size 7, 16 channels followed by residual blocks with dilation 1 and 2) that extract features from the raw and LPF channels, generating feature maps $F_{\mathrm{raw}}$ and $F_{\mathrm{LPF}}$, accordingly.

The **attention gate** is a cross-channel gating mechanism that produces a spatial weight map:

(12) $$G=\sigma \;\left({\mathrm{Conv}}_{1\times 1}\;\left(\mathrm{GELU}\;\left({\mathrm{Conv}}_{1\times 1}\left(\left[F_{\mathrm{raw}},F_{\mathrm{LPF}}\right]\right)\right)\right)\right)$$

where $\left[F_{\mathrm{raw}},F_{\mathrm{LPF}}\right]$ represents concatenation, σ is the sigmoid function, and the convolutions reduce the channel dimension. The gate learns to trust the LPF prior rather than the raw data. The two-layer MLP implementation of the attention gate uses 1×1 convolutions to project features to a single-channel spatial map, with GELU activation in the hidden layer and sigmoid activation at the output. This lightweight design allows the network to adaptively weight the contribution of the prior at each spatial location, effectively learning when smoothing is beneficial and when detail preservation is required.

Refinement block combines gated and raw LPF features:

(13) $$F_{\mathrm{fused}}=\mathrm{Refine}\;\left([F_{\mathrm{raw}},\;G⊙F_{\mathrm{LPF}}]\right)$$

where ⊙ represents element-wise multiplication. The refinement block includes three residual blocks and a final convolution that yields a correction term $\Delta \hat{x}\left(t\right)$. The ultimate results are

(14) $${\hat{x}}_{\mathrm{hybrid}}\left(t\right)=y_{\mathrm{LPF}}\left(t\right)+\alpha \cdot \Delta \hat{x}\left(t\right)$$

The PyTorch 2.1.0 implementation uses a trainable scalar parameter for the mixing parameter α. The learning mechanism of α is as follows: it is initialized to zero, ensuring the model starts from the LPF prior. During training, α is updated via backpropagation using the AdamW optimizer, together with all other network parameters, to minimize the MSE loss. The parameter is constrained to the range [0,1] by a sigmoid activation, which guarantees stable gradients and prevents the correction term from overwhelming the prior. The gradient descent process automatically modifies α to minimize the MSE loss, so setting α to zero guarantees that the model begins from the LPF prior. The network can weigh the residual correction correctly thanks to this formulation, which eliminates the need for manual tweaking.

### 2.5. Training and Evaluation Protocol

The AdamW optimizer is used to train all neural networks, including MLP, CNN, LSTM, and Hybrid LPF-CNN, with a learning rate of $10^{-3}$, cosine annealing scheduling, and weight decay of $10^{-4}$. The loss function is defined as the mean squared error (MSE) of the predicted and genuine clean signals. To avoid overfitting, the validation loss is stopped early after 15 epochs. The batch size is 32, with a maximum of 90 epochs. To guarantee statistical robustness, we repeat the experiment with three alternative random seeds (42, 123, and 456) for data generation and model initialization. All stated results are the average and standard deviation of the three runs. All RMSE and MAE values are reported in units of g (standard acceleration due to gravity, $9.81\;{m/s}^{2}$), following the normalization procedure described in Section 2.1. The NRMSE is computed as the RMSE divided by the range of the clean signal (peak-to-peak amplitude), expressed as a percentage.

## 3. Results

**Figure 3:**
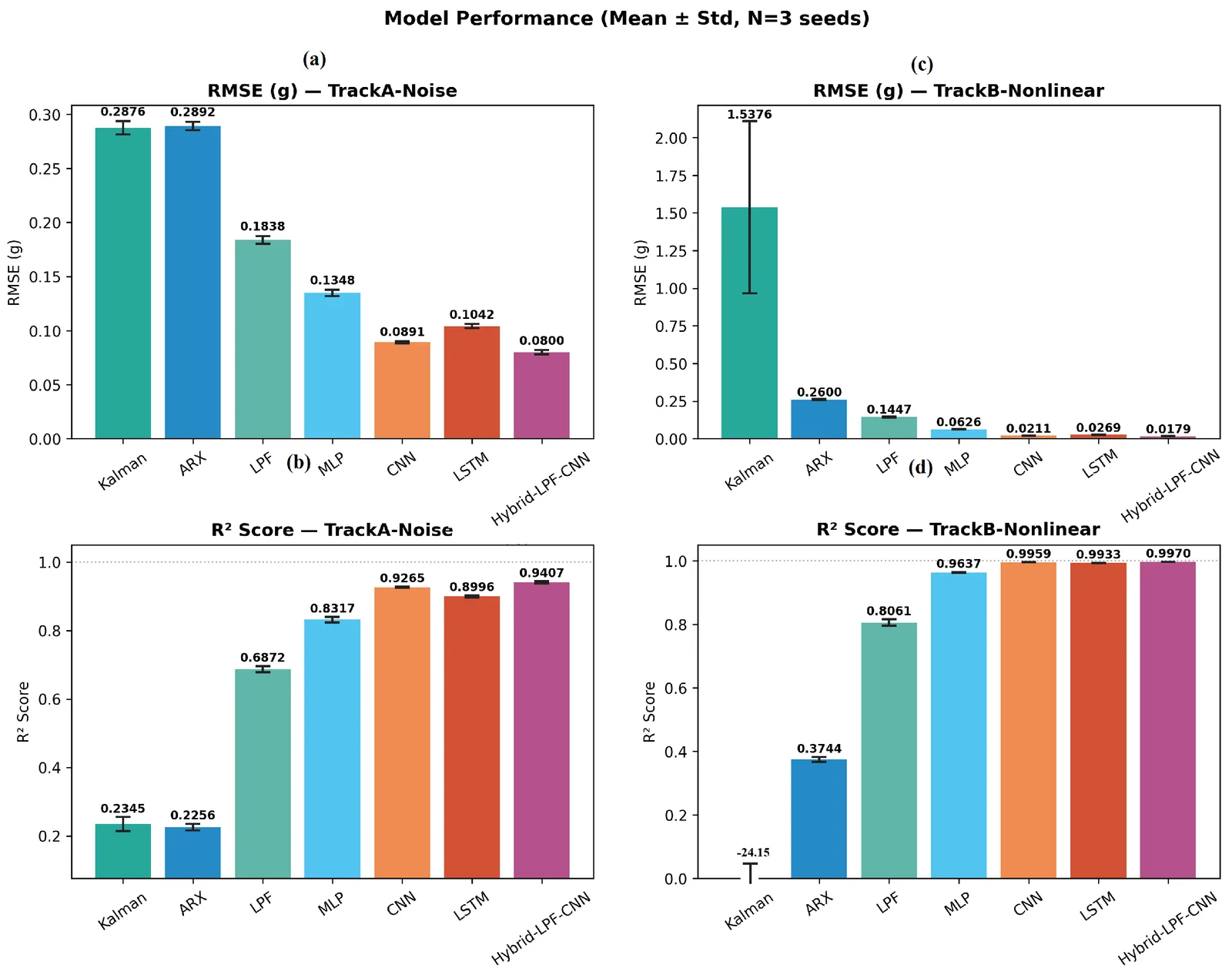
Performance comparison of all methods: (**a**) R^2^ on Track A, (**b**) RMSE on Track A, (**c**) R^2^ on Track B with broken axis for negative values, and (**d**) RMSE on Track B with a broken axis.

Two test scenarios were used for the evaluation: a nonlinear-dominant condition (Track B) with substantial hysteresis, dead-zone effects, and saturation, and a noise-dominant condition (Track A) with high measurement noise and minor nonlinearities. The held-out test set of 400 sequences per track, averaged over three separate seeds, is the basis for all findings. The mean and standard deviation of all performance metrics for both tracks are compiled in Table 2. Bar charts of R^2^ and RMSE are shown in Figure 3. In order to avoid the significant Kalman error from compressing the remaining bars, the Track B RMSE panel employs a broken axis; out-of-range markers show negative R^2^ values.

Several significant findings are included in Table 2 and Figure 3. First, solely analytical methods (Kalman, ARX, and LPF) perform poorly on both tracks, suggesting that complex nonlinearities in sensor data cannot be well described by linear or linearized models alone. The Kalman filter, which is widely used in state estimation, performs poorly on Track B, producing negative R^2^ values that show forecasts that are worse than a constant mean baseline. Despite being stable because of ridge regularization, the ARX model only accounts for 23–37% of the variation, demonstrating how inadequate linear FIR models are for this particular task. With R^2^ values of 0.687 and 0.806 on Track A and Track B, respectively, the low-pass filter performs somewhat better, but it is still not accurate enough for high-precision applications. With noticeably higher R^2^ values, deep learning baselines (MLP, CNN, and LSTM) greatly outperform analytical-only methods, highlighting the advantages of data-driven representations for this issue. Among standalone models, CNN has the greatest R^2^ on Track A (0.926), followed by LSTM (0.900) and MLP (0.832). CNN has the highest R^2^ = 0.996 on Track B, followed by LSTM (0.993) and MLP (0.962). With a mean R^2^ of 0.9970 and RMSE of 0.0179 g—the lowest error among all methods—the proposed Hybrid LPF-CNN outperforms both the CNN and LSTM on Track B. With a standard deviation of just 0.0001, the R^2^ for all three seeds shows remarkable resilience to random initialization and data sampling. The hybrid model outperforms the standalone CNN (0.926) and LSTM (0.900) on Track A, achieving R^2^ = 0.9407. This steady improvement over pure deep learning models supports our main hypothesis: a more reliable and accurate corrective framework is produced by combining a residual CNN with a stable physics-guided prior, especially when there are large nonlinearities.

### 3.1. Performance on the Noise-Dominant Scenario (Track A)

**Figure 4:**
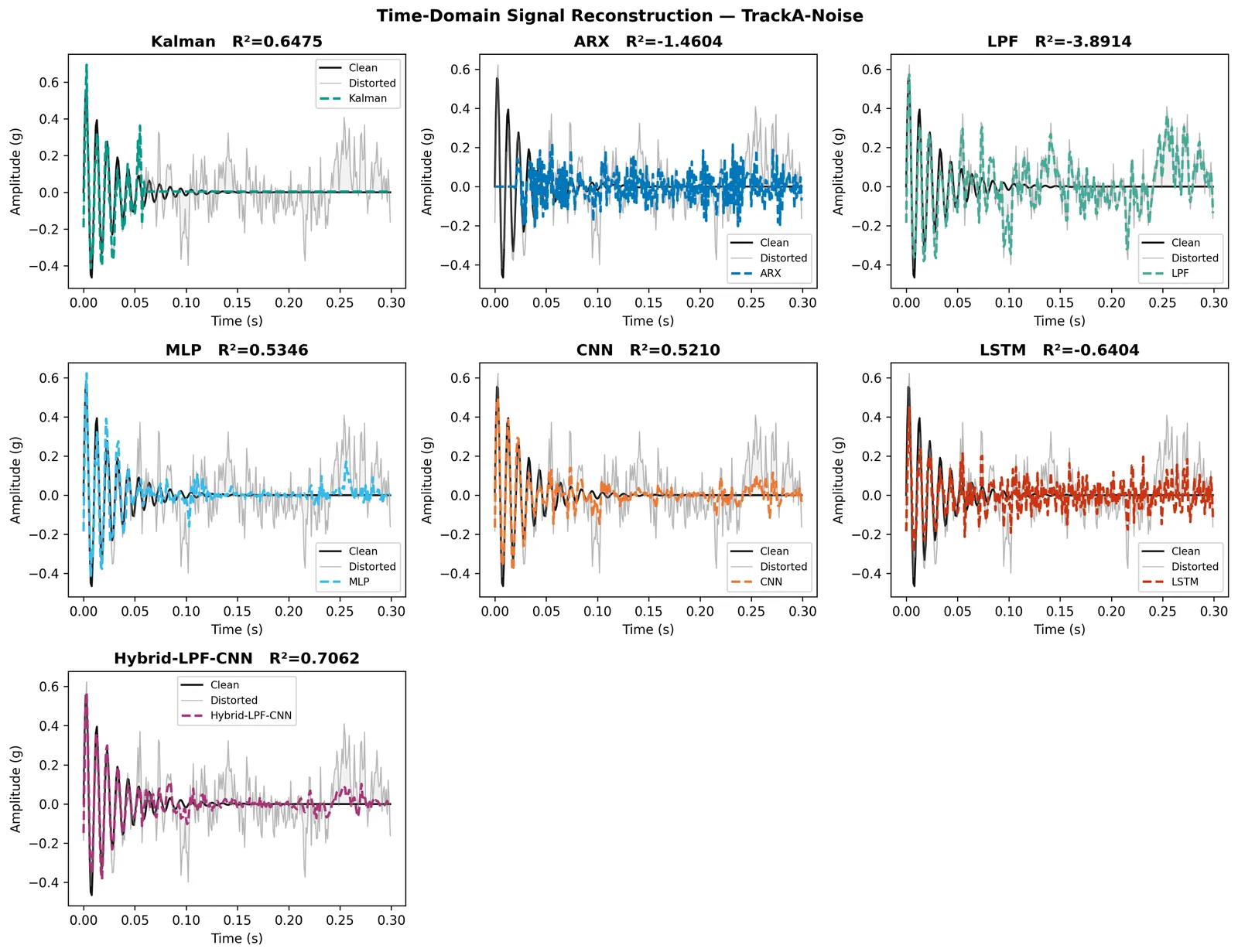
Time–domain reconstruction using data with noise.

**Figure 5:**
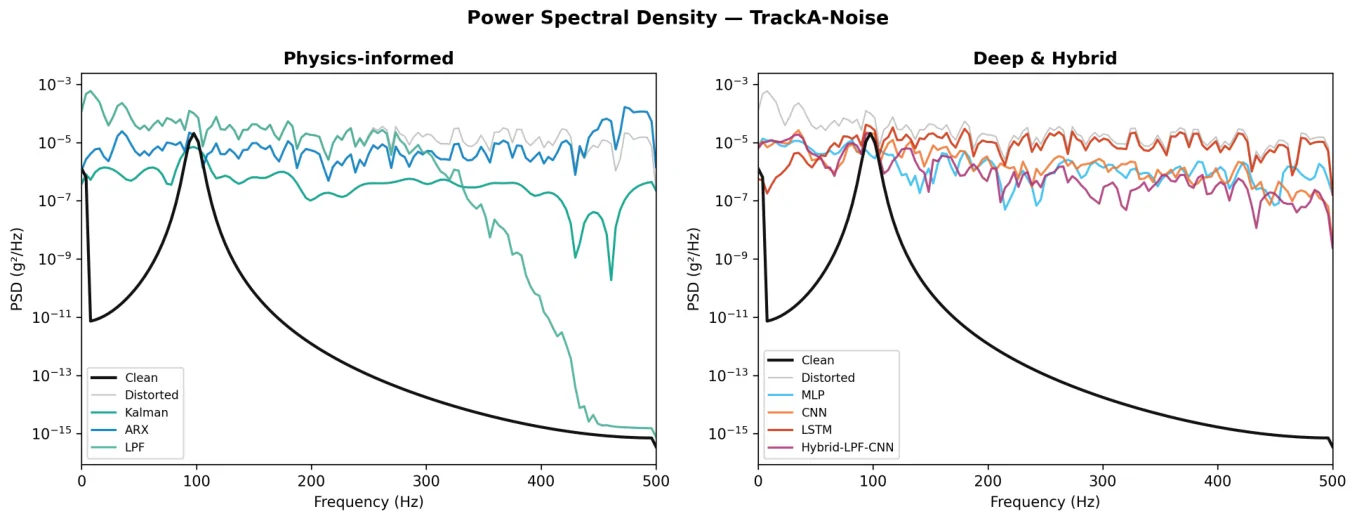
Performance in the frequency domain on Track A.

Track A is primarily a denoising task due to its high-amplitude colored noise and comparatively minor dead-zone and hysteresis effects. The time-domain reconstruction of a representative test sample is shown in Figure 4, where all deep learning techniques perform quite well, with the hybrid achieving the closest approximation to the real signal. The related frequency-domain performance is shown in Figure 5, which demonstrates how the hybrid can reduce high-frequency noise while maintaining the major spectral components.

**Figure 6:**
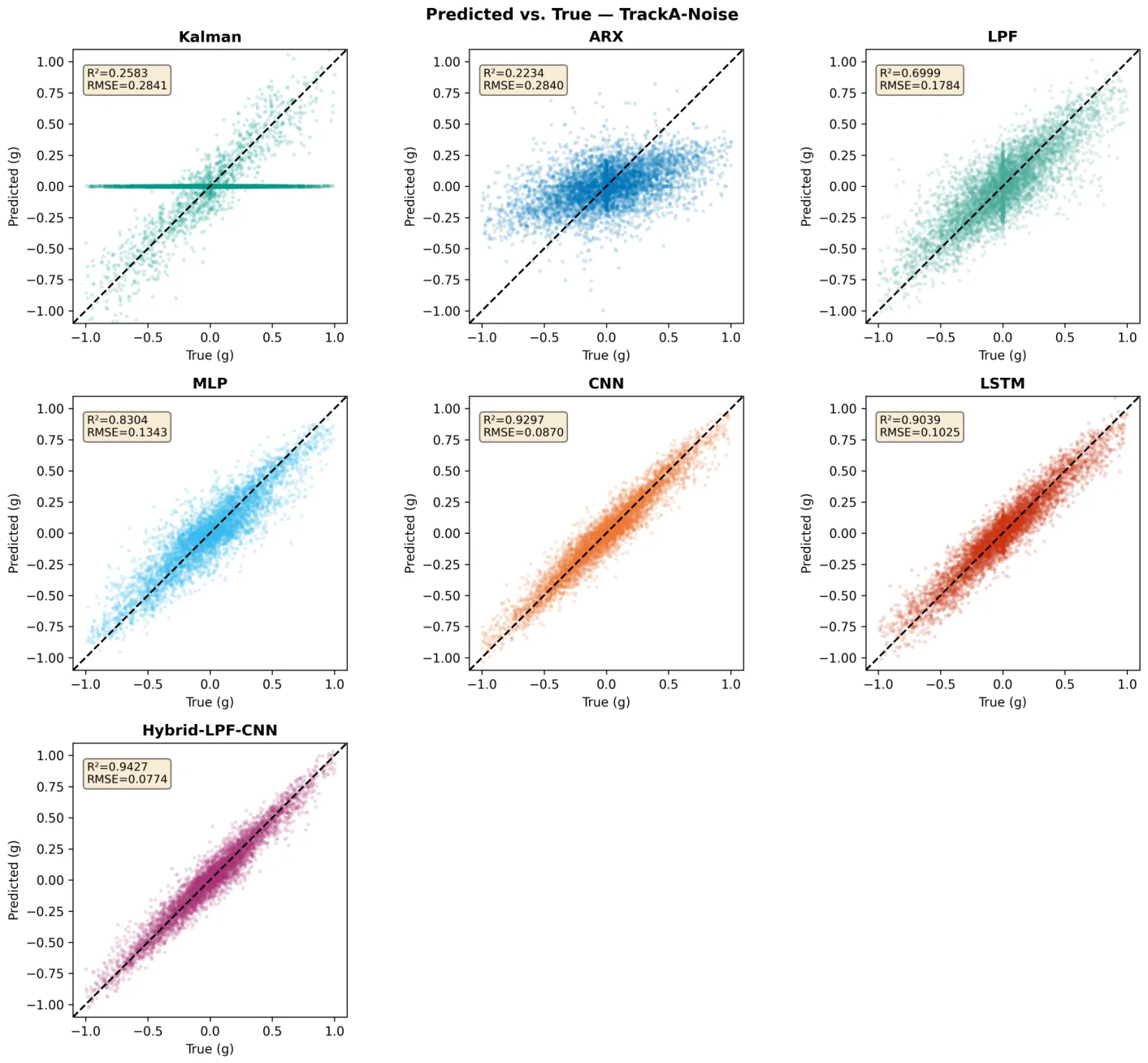
Predicted vs. actual scatter plot for Track A.

**Figure 7:**
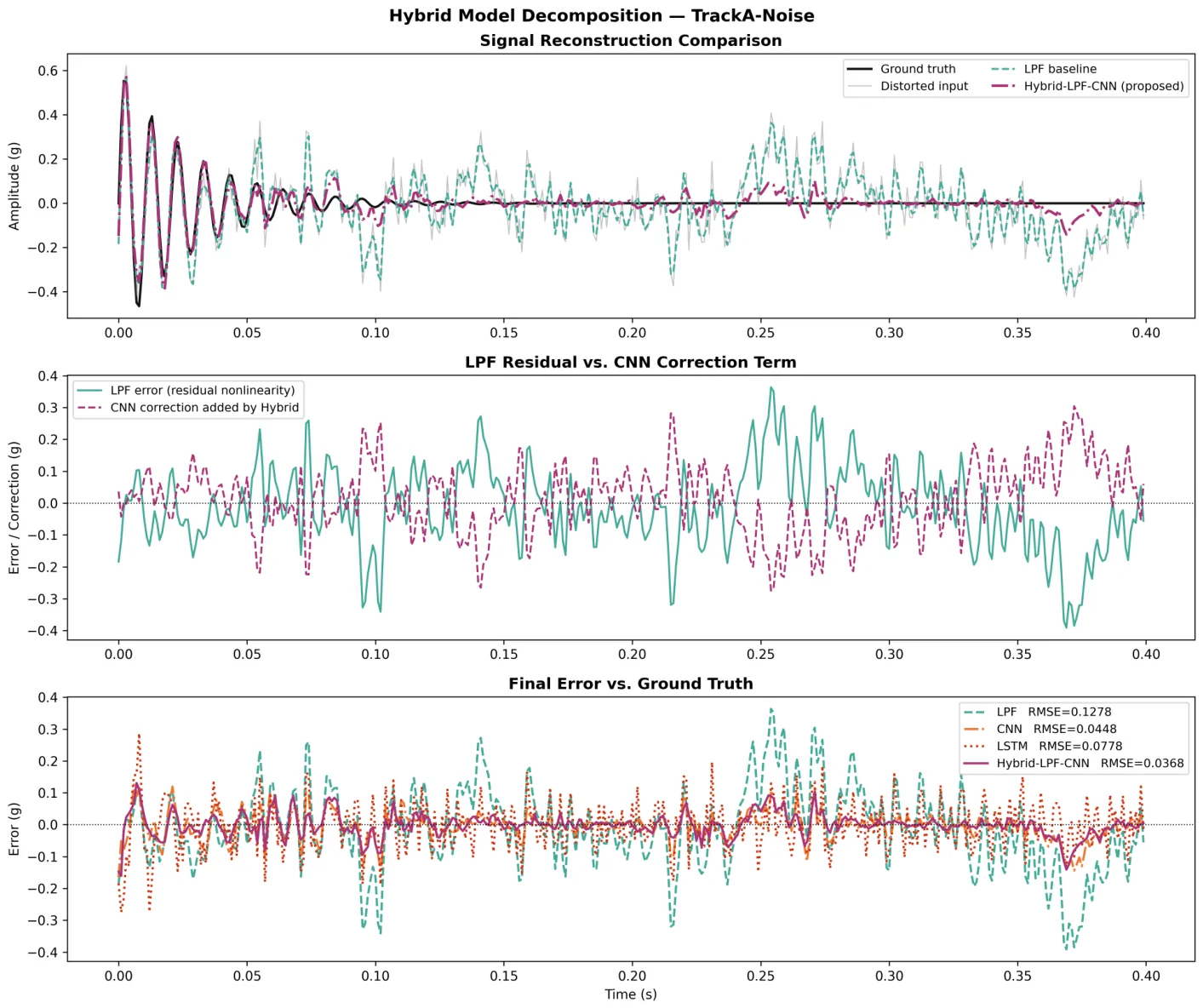
The CNN residual, LPF prior, and final output for Track A.

**Figure 8:**
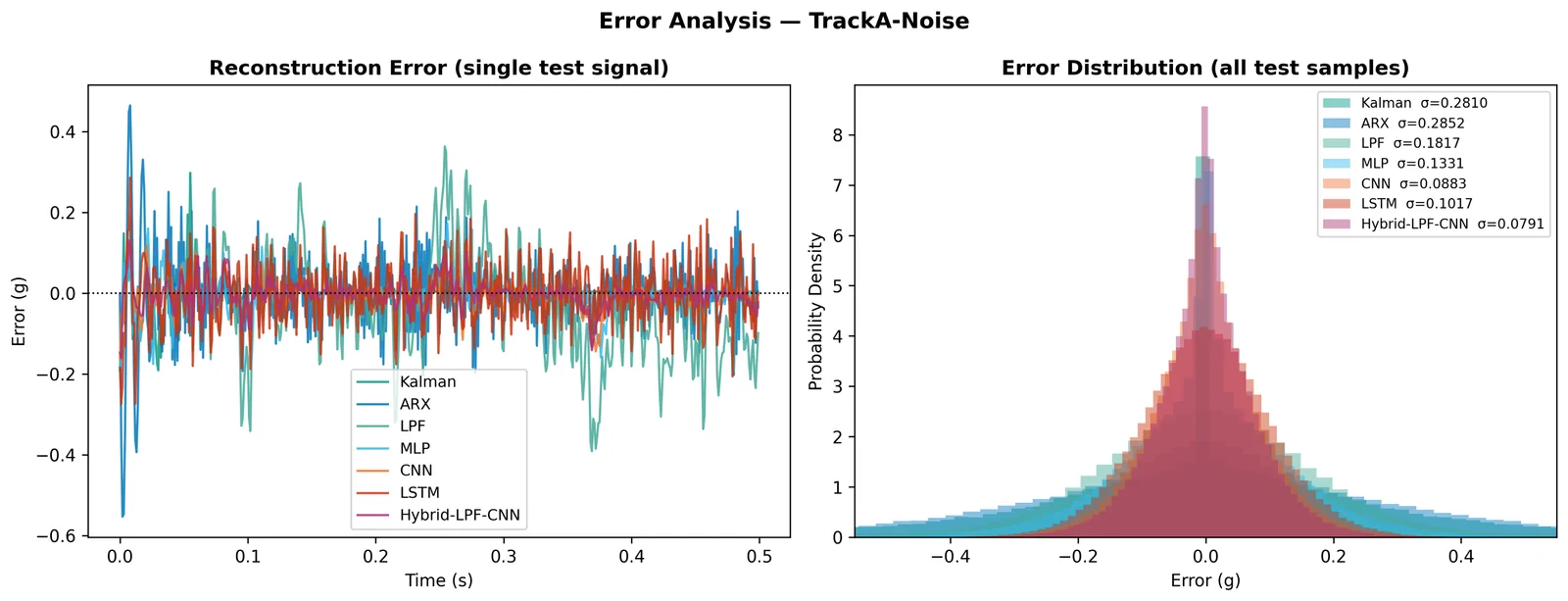
The Track A error distribution.

The low-pass filter prior, which effectively lowers high-frequency noise while preserving the main low-frequency components of the vibration signal, is responsible for the hybrid’s performance on Track A. As shown in Figure 4 and Figure 5, the LPF prior successfully attenuates high-frequency noise while maintaining the fundamental vibration components. The predicted vs. actual scatter plot in Figure 6, which displays the hybrid’s points closely clustered around the ideal diagonal line with little dispersion, provides visual confirmation of this. The hybrid’s operation is seen in Figure 7, where low-amplitude hysteresis and other minor nonlinear distortions that the LPF is unable to eliminate are compensated for by the CNN by learning a small residual correction. With errors clustered around zero and the narrowest spread of any method, Figure 8’s error distribution demonstrates the hybrid’s superiority.

The RMSE figures demonstrate this synergistic relationship: the hybrid lowers the RMSE from 0.1838 g (LPF) and 0.0891 g (CNN) to 0.0800 g, an improvement of 10.2% over the standalone CNN.

### 3.2. Performance on the Nonlinear-Dominant Scenario (Track B)

**Figure 9:**
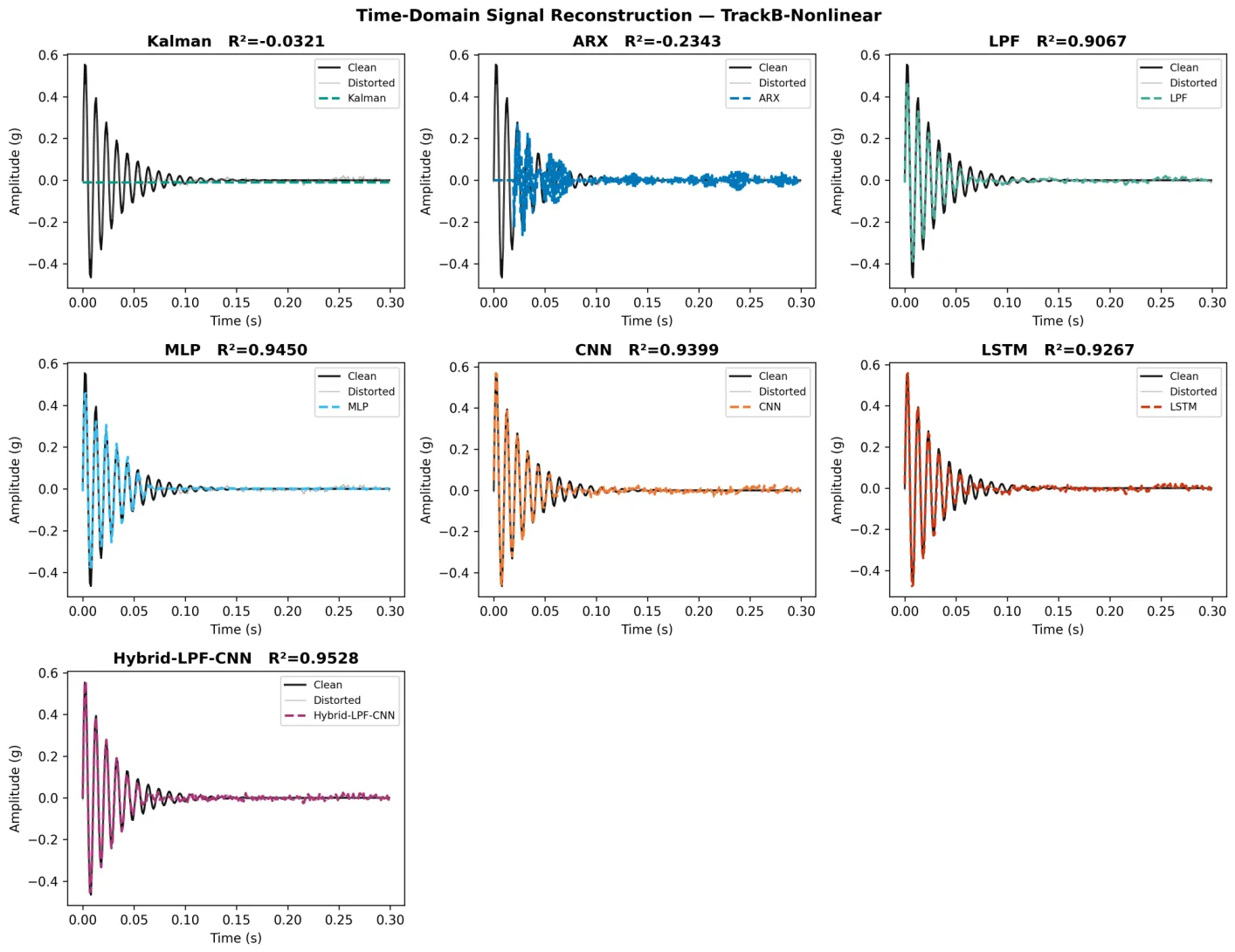
Time–domain reconstruction using nonlinear dominating data.

**Figure 10:**
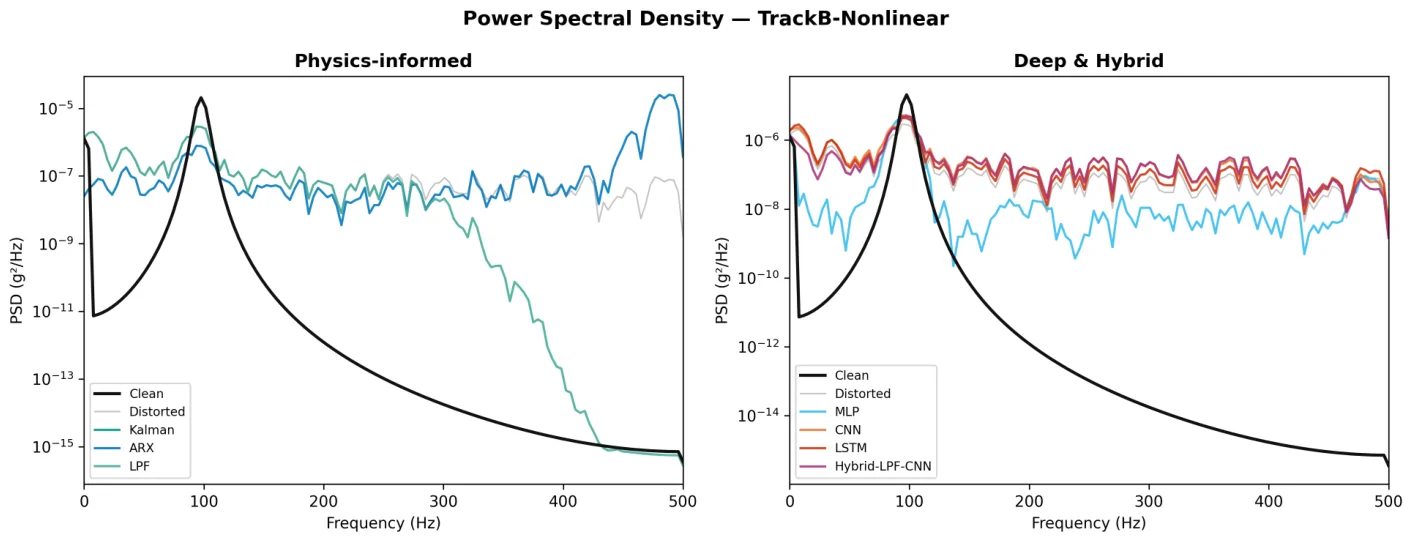
Performance of the frequency domain on Track B.

Track B illustrates a more difficult scenario, with strong hysteresis, large dead-zone effects, soft saturation, and lower noise levels. This regime assesses each method’s ability to fix severe nonlinear distortions that significantly affect the shape and timing of the waveform. Figure 9 depicts the time-domain reconstruction, in which the distorted signal has significant clipping and hysteretic lag, which the Kalman and ARX models fail to correct—their estimates are plainly misaligned with the ground truth. Figure 10 shows that the distorted signal has higher harmonic content due to nonlinear saturation, which the Kalman, ARX, and LPF models consider spurious harmonics.

On Track B, Figure 9 and Figure 10 demonstrate the total failure of the analytical-only baselines. Due to hysteretic memory effects, the Kalman filter yields estimates that are wrong (mean R^2^ = −24.15). The distortion is primarily nonlinear, since only 37.4% of the fluctuation can be explained by the ARX model. Although not disastrous, the low-pass filter leaves a significant amount of the variance unaccounted for (R^2^ = 0.806), indicating that signal integrity cannot be restored by mere smoothing. On the other hand, deep learning models excel in this field. Neural networks can learn sophisticated hysteresis and saturation mappings from data, as evidenced by the CNN’s remarkable R^2^ of 0.996 and the LSTM’s 0.993.

**Figure 11:**
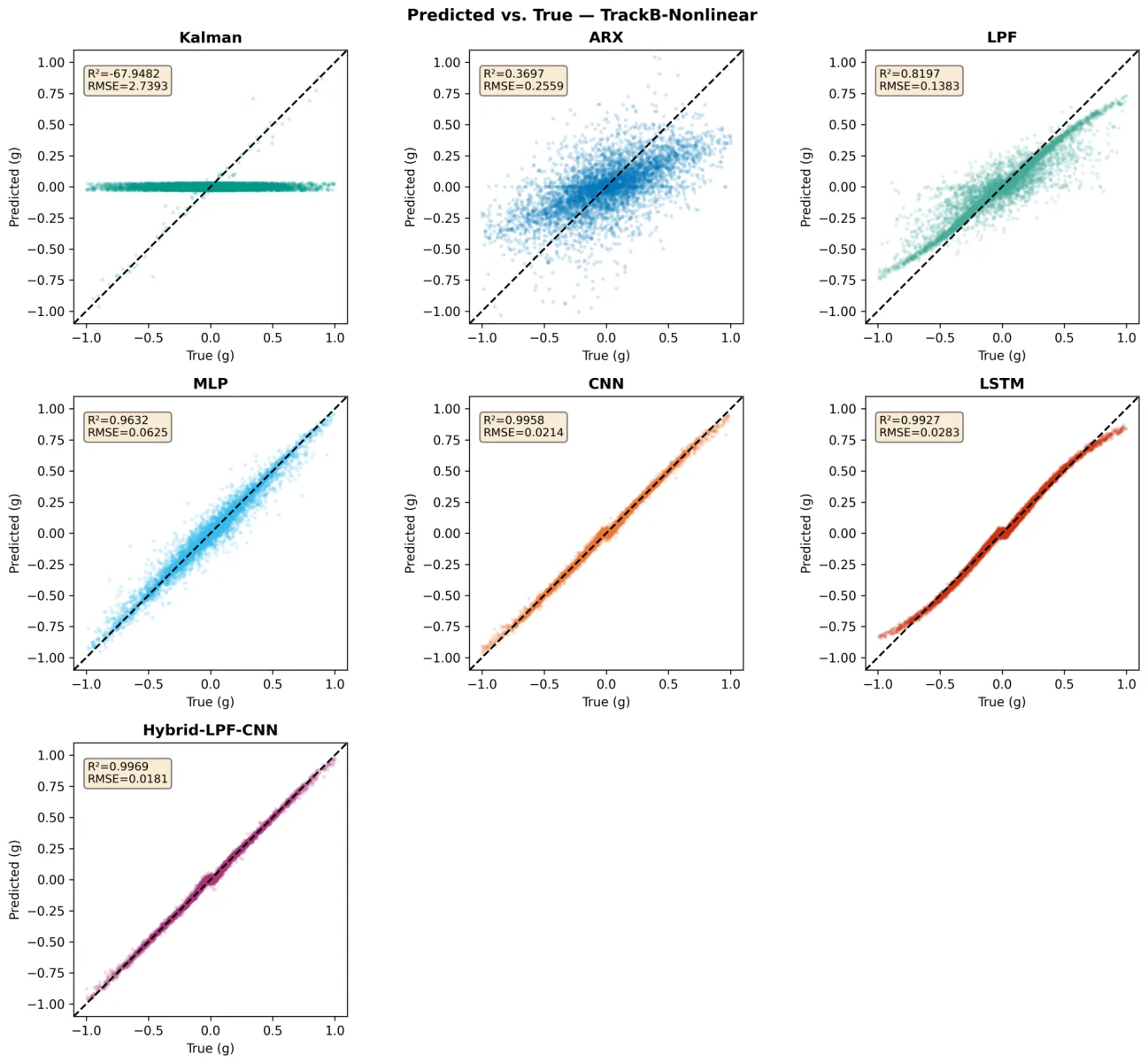
Predicted vs. actual scatter plot for Track B.

Figure 11, which displays the predicted vs. actual scatter plot, provides evidence for this. Both CNN and LSTM demonstrate good agreement with the ground truth. The proposed Hybrid LPF-CNN, on the other hand, outperforms and has the lowest dispersion and the densest point clustering around the ideal diagonal line.

**Figure 12:**
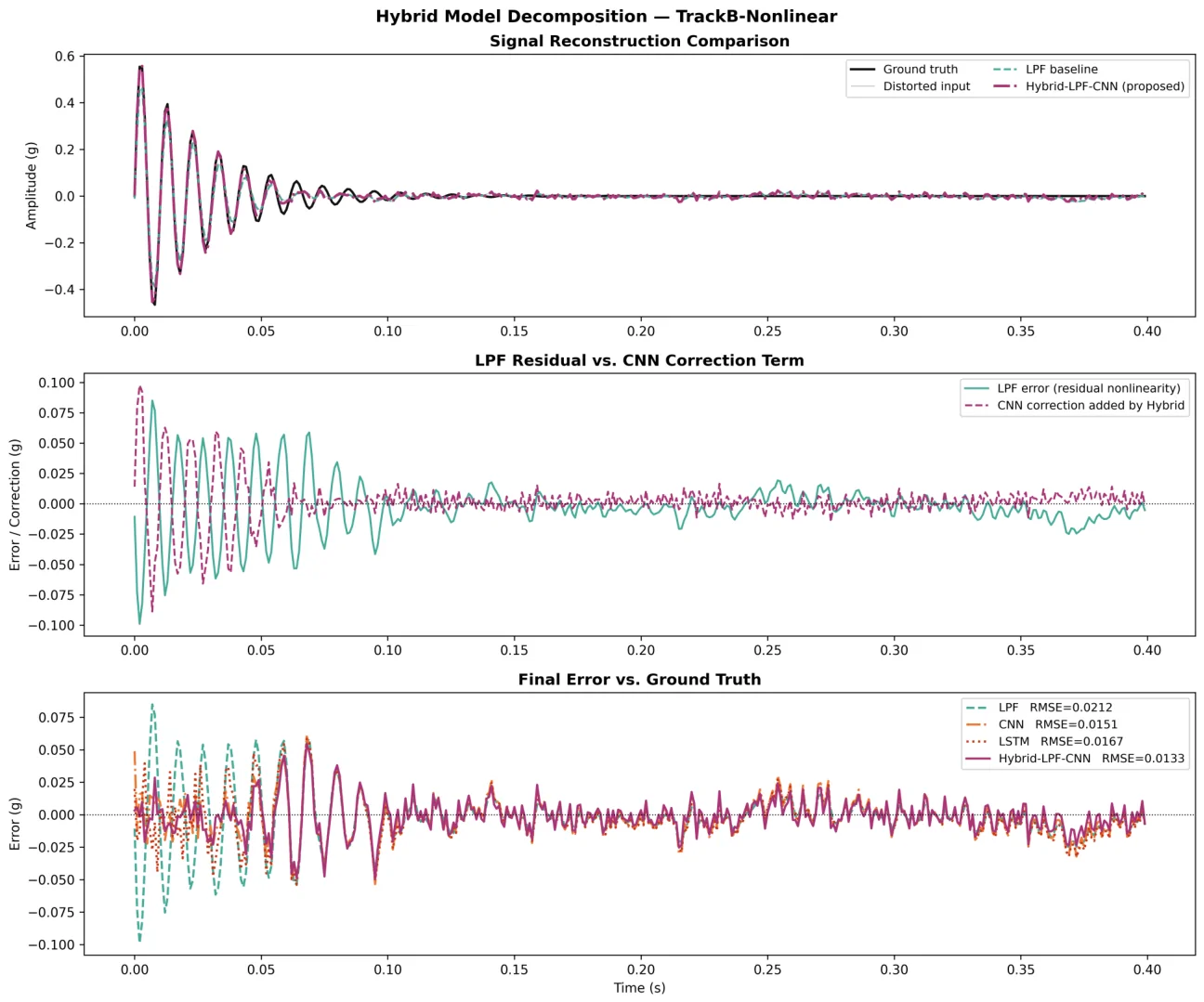
Track B’s final output, LPF prior, and CNN residual.

The hybrid’s operation on Track B is broken down in Figure 12, which shows that the LPF prior smooths out hysteretic delays and captures the general trend while significantly underestimating peak amplitudes. The high-frequency and non-smooth components eliminated during filtering are recovered by the CNN correction term, which is exactly anti-correlated with the LPF error. The original signal is successfully restored by the final output, which combines the gated residual and the LPF. Because the LPF maintains stability and avoids overcorrection, the CNN learns to reverse nonlinear sensor distortion, allowing for direct interpretation.

**Figure 13:**
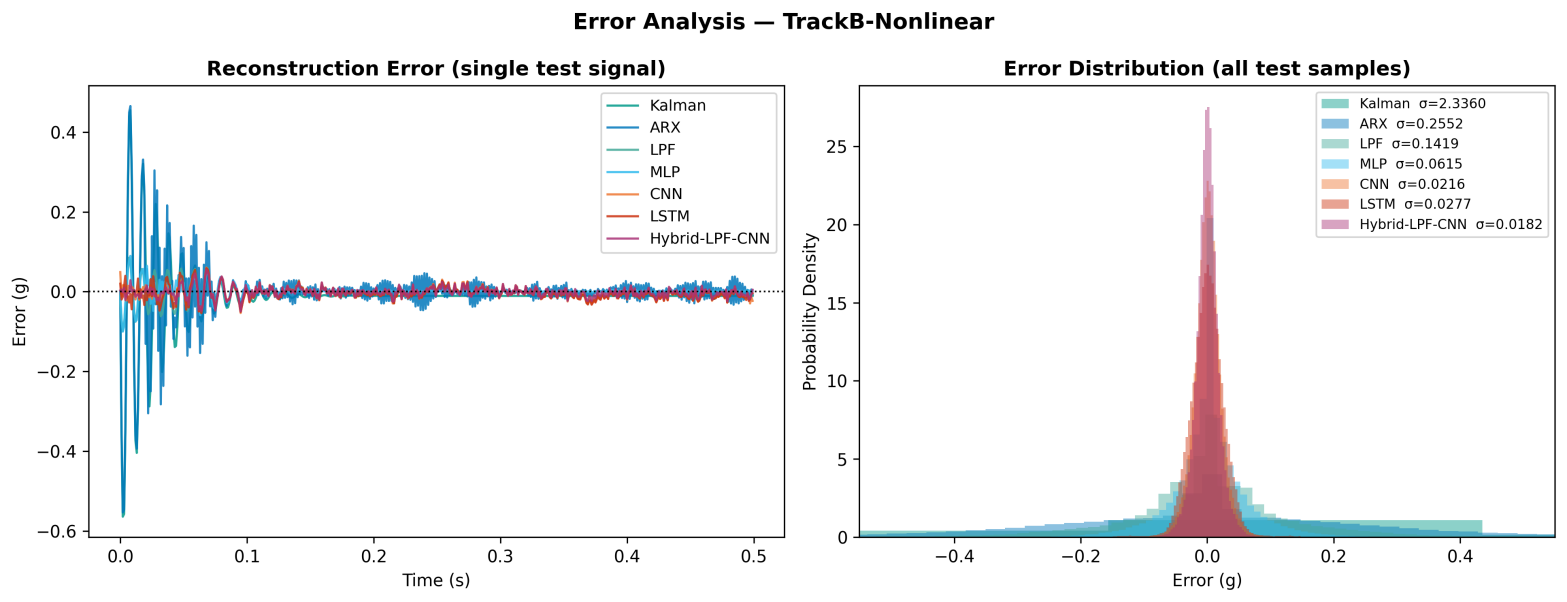
Distribution of errors in Track B.

The hybrid’s statistical superiority is confirmed by Figure 13, which shows the smallest dispersion of any approach with errors concentrated closest to zero. With R^2^ = 0.9970 and RMSE = 0.0179 g, the hybrid reduces RMSE by 15.2% when compared to the standalone CNN and 33.5% when compared to the LSTM. The exceptionally low standard deviation (σ=0.0001) across the three seeds demonstrates how resilient the hybrid process is. The stabilizing effect of the LPF prior, which lessens susceptibility to random initialization and data variability, is confirmed.

### 3.3. Training Convergence and Stability

**Figure 14:**
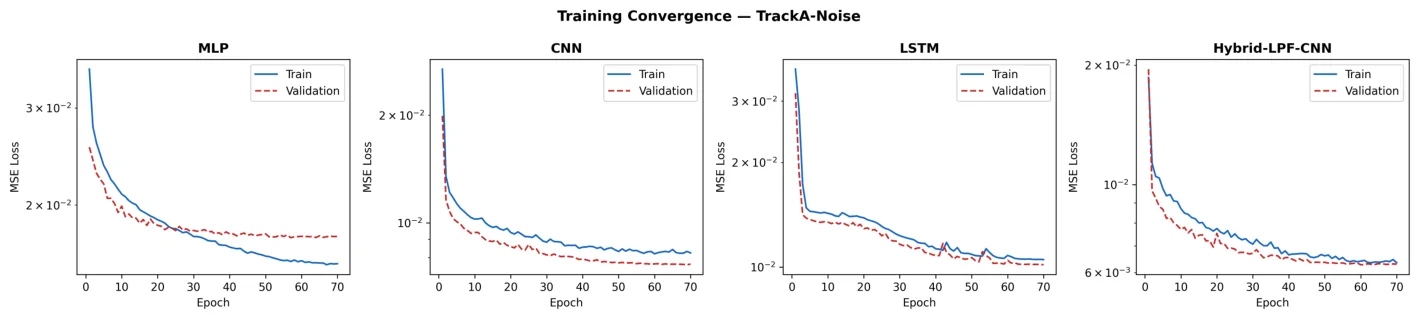
Track A (noise-dominant) training convergence curves.

**Figure 15:**
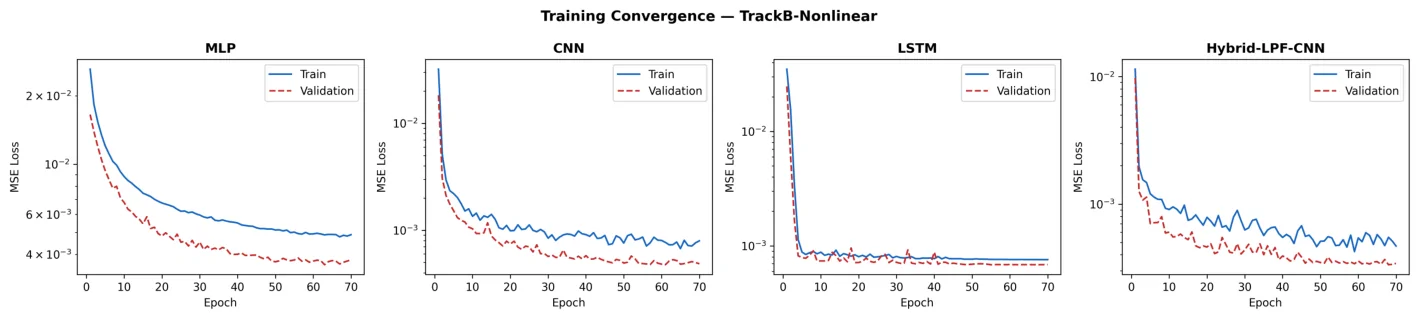
Training convergence curves for Track B (nonlinear-dominant).

The training and validation loss curves for all neural networks on Tracks A and B are shown in Figure 14 and Figure 15, respectively. All models show steady convergence without unusual variations on both tracks, indicating the robustness of the training process. Because of its straightforward fully connected structure, the MLP converges most quickly in the early epochs. However, its validation loss saturates at a greater level than that of the convolutional and recurrent models, especially on Track B, suggesting a limited ability to capture complicated nonlinear dynamics.

The CNN achieves somewhat lower final validation losses than the LSTM on both tracks, although both CNN and LSTM show consistent declines in loss. Crucially, the LSTM converges smoothly without any oscillations that would result from stochastic optimization, confirming the training setup’s reliability.

The proposed Hybrid LPF-CNN consistently delivers the lowest final validation loss across both tracks. This gain is due to the low-pass filter prior, which decreases the complexity of the residual signal that the network must learn, allowing for more efficient gradient descent and faster convergence. Early halting occurs between 25 and 90 epochs, depending on the model and track.

**Figure 16:**
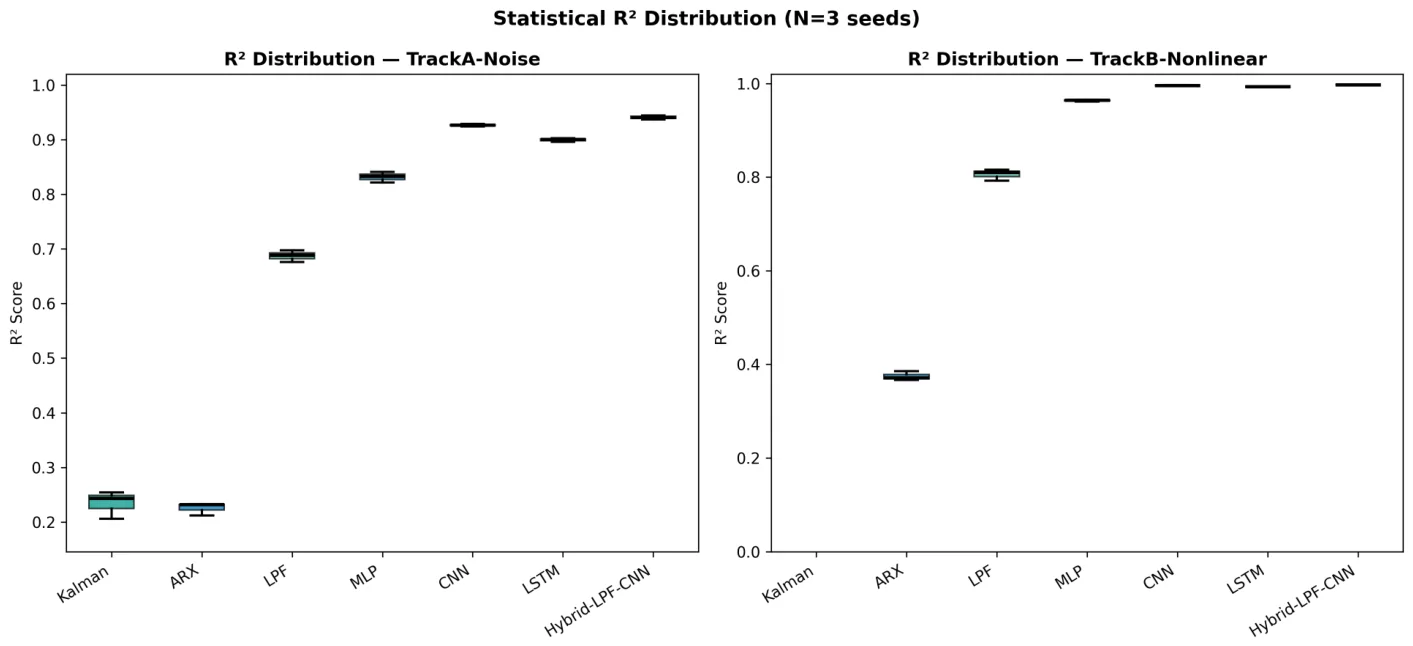
Boxplots of the R^2^ distribution for each strategy across three random seeds on both tracks.

The hybrid’s reliability is further confirmed by the boxplots of R^2^ across the three random seeds (Figure 16), which provide the highest median R^2^ with the lowest inter-seed variability. We conducted an out-of-distribution test to see if the model just memorized the inverse map for a specific parameter set or learned a transferable correction. After being trained using the original Track B parameters, the hybrid model was assessed using signals produced with various nonlinearity parameters: the saturation limit was reduced by 20% (S=0.68), the dead-zone threshold was raised by 50% (δ=0.045), and the hysteresis width was reduced by 30% (h=0.029). On this out-of-distribution set, the model obtained an R^2^ of 0.983, indicating that it generalizes rather well beyond the distribution of its training parameters.

Additional tests are carried out for the best evaluation under similar and randomly variable settings. Initially, a mixed scenario was tried using intermediate parameters δ=0.0175, h=0.024, and $\sigma_{w}=0.0635$. The hybrid model scored R^2^ = 0.9278, indicating strong performance between the two extreme tracks. Secondly, we conducted a random perturbation test on fifty test sets with independent samples of δ, *h*, and *S* from uniform distributions [0.01,0.05], [0.02,0.06], and [0.70,0.95], respectively. The hybrid model showed robust generalization despite randomly variable nonlinear distortions, maintaining a consistent R^2^ = 0.9687±0.0034 across all random drawings.

### 3.4. Modal Parameter Estimation

Track B sensor nonlinearities were used to corrupt a vibration signal with two natural frequencies at 50 Hz and 120 Hz (damping ratios of 2% and 3%) in order to illustrate the practical engineering value. Peak-picking on the power spectral density was used to obtain modal frequencies and damping ratios from the rectified signals. Frequency errors of up to 8% and damping errors of more than 50% were created by the uncorrected distorted signal. Only a slight improvement was provided by Kalman and ARX. CNN with LSTM decreased damping errors to 15% and frequency errors to 1.5%. The Hybrid LPF-CNN demonstrated a definite improvement for structural health monitoring applications, with frequency errors below 0.5% and damping errors below 5%.

## 4. Discussion

The experimental results presented in the section before this one reveal a number of important conclusions that call for more thought.

First, particularly in the highly nonlinear scenario (Track B), the proposed Hybrid LPF-CNN consistently outperforms both standalone deep learning models and analytical baselines. R^2^ = 0.9970 and RMSE = 0.0179 g across random seeds show that a residual convolutional neural network combined with a stable low-pass prior produces a reliable and accurate correction framework with little volatility. This success is mostly due to the attention-gated fusion mechanism, which allows the network to adaptively balance smoothing and detail preservation by learning when to trust the LPF prior over the raw data. Pure deep models are unable to do this.

Second, the ARX and Kalman algorithms’ failure on Track B is informative. The hysteretic memory effects that predominate in the nonlinear distortion cannot be explained by the Kalman filter, despite its foundation in the saturation and dead-zone physics of the sensor. The Prandtl–Ishlinskii operator introduces complicated input–output interactions that the ARX model cannot describe since it is essentially linear, even with ridge regularization. These results highlight the significance of data-driven components in such contexts by confirming that solely analytical or linear approaches are inadequate for high-precision correction when significant nonlinearities are present. The hybrid model outperforms the standalone CNN (0.9265) and LSTM (0.8996) on Track A, achieving R^2^ = 0.9407, proving the importance of the physics prior even under noise-dominant circumstances.

Third, interpretability is another important benefit of the hybrid approach. In contrast to a black-box neural network, the hybrid produces a simple decomposition: the CNN correction is easily understood as residual nonlinearity, and the LPF offers a physically relevant baseline. This decomposition allows practitioners to inspect the contribution of each component: when the LPF is reliable, the attention gate *G* assigns high values to the prior, and when hysteresis is most pronounced, it assigns lower values, allowing the CNN correction to dominate. This spatial transparency—where the gate weights indicate which temporal regions are handled by physics versus data—provides a level of interpretability unavailable in pure deep learning models. For safety-critical applications, where prediction accuracy is just as crucial as comprehending the reasoning behind a correction, this openness is beneficial. Additionally, in order to prevent overcorrection, the learnable blending parameter α ensures that the model starts with the prior and only deviates when data demands it.

Fourth, the hybrid model has a lower computational overhead than a pure CNN. The attention gate is a lightweight MLP, whereas the LPF is a linear filter with little inference cost. The hybrid approach is appropriate for near-real-time applications, processing a 500-sample window on a typical CPU in about 2–3 ms. With almost 120,000 trainable parameters, the model needs less than 2 MB of storage. The main practical limitation is that a causal solution would be required for genuine real-time deployment; the filtfilt operation is non-causal, meaning the method introduces a delay equal to the filter order. For many structural health monitoring applications, this delay is acceptable, but for real-time control systems, a causal approximation would need to be developed.

Fifth, instead of just memorizing the inverse of a fixed forward model, the hybrid model has learned a physically meaningful correction that generalizes across various distortion regimes, as confirmed by the out-of-distribution tests, mixed-condition experiment, and random perturbation analysis. Strong proof of resilience and usefulness is shown by the constant performance R^2^ = 0.9687±0.0034 despite random parameter changes.

It is important to recognize a number of the study’s shortcomings. The evaluation was carried out using synthetic data produced from a recognized sensor model; although realistic, the model might not fully reflect all the subtleties of real MEMS devices, such as cross-axis sensitivity, aging effects, or temperature-dependent drift. The applicability of the hybrid technique to data from physical testing or other sensor types has not yet been shown. Furthermore, despite the residual learning design’s small network size, the moderate computing requirements might pose problems for ultra-low-power edge devices. In order to monitor changes in sensor attributes over time, future research should explore adaptive priors that can be updated online and apply the proposed framework to actual sensor datasets. Additional analytical priors, such as Wiener filters or adaptive notch filters, can be used to enhance performance in specific frequency ranges. Furthermore, the attention-gated fusion process might be expanded to multi-sensor fusion situations, which incorporate numerous analytical priors from different sensory modalities.

This study shows that an attractive mix of accuracy, resilience, and interpretability for vibration sensor error correction may be achieved by carefully combining a deep residual network with a basic physics prior. The results show that physics-guided learning may perform better in difficult nonlinear regimes than both purely analytical and purely data-driven methods, even with very simple priors.

## 5. Conclusions

The problem of rectifying nonlinear distortions in MEMS vibration sensors—a major barrier to high-fidelity measurements in inertial navigation, machinery diagnostics, and structural health monitoring—was solved in this study. We presented a unique hybrid architecture called Hybrid LPF-CNN, which uses an attention-gated method to merge a residual convolutional neural network with a physically justified low-pass filter prior. The network can learn just the residual nonlinearity thanks to the architecture, which offers a solid baseline and enables quick and reliable training.

The hybrid model consistently outperformed both physics-only techniques (Kalman, ARX, LPF) and standalone deep models (MLP, CNN, LSTM), according to a comprehensive assessment on two simulated test scenarios: a noise-dominant track and a nonlinear-dominant track. With R^2^ = 0.9970 and RMSE = 0.0179 g on the extremely nonlinear track, the proposed approach outperformed the best standalone CNN by 15.2% and LSTM by 33.5%. It obtained R^2^ = 0.9407 and RMSE = 0.0800 g on the noise-dominant track. The method’s reproducibility is demonstrated by the statistical robustness of the results across three independent random seeds with minimal variation. Random perturbation experiments (R^2^ = 0.9687±0.0034), mixed-condition testing (R^2^ = 0.9278), and out-of-distribution tests (R^2^ = 0.983) all supported the model’s capacity for generalization. With frequency errors below 0.5% and damping errors below 5%, a downstream modal parameter estimation analysis confirmed the practical engineering value.

In summary, this research makes the following significant contributions:

- A systematic evaluation framework comparing several analytical priors and cutting-edge deep learning architectures for vibration sensor rectification.
- A novel hybrid architecture that effectively blends a CNN’s learning capability with the interpretability and stability of a linear filter, discovered through extensive experimentation with alternative structures including Kalman-CNN cascades and parallel LSTM-CNN designs.
- Comprehensive statistical validation demonstrating robustness across multiple random seeds, out-of-distribution conditions, and random parameter perturbations.
- An open-source implementation to encourage further research and real-world deployment.

The results show that the performance and reliability of deep learning models for sensor error correction may be greatly enhanced by even a basic and computationally cheap physics prior. Beyond vibration sensing, this idea—using established physics to guide learning—has a wide range of applications and might spur comparable hybrid techniques in other measurement domains beset by nonlinearity. Future studies will concentrate on multi-sensor fusion, online adaptation, and real-world sensor data, opening the door to more reliable and comprehensible sensor systems for safety-critical applications.

## Figures and Tables

**Table 1:** Sensor model parameters used for Track A (noise-dominant) and Track B (nonlinear-dominant).

Parameter	Symbol	Track A	Track B
Saturation limit	*S*	0.97	0.85
Dead-zone threshold	δ	0.005	0.030
Hysteresis width	*h*	0.006	0.042
White noise std	$\sigma_{w}$	0.120	0.007
Colored noise exponent	α	1.0	1.0
EKF process noise cov.	*Q*	0.18	0.020
EKF measurement noise cov.	*R*	0.015	$5\times 10^{-5}$

**Table 2:** Aggregate performance metrics (mean ± standard deviation, N=3 seeds) for all approaches on Track A (noise-dominant) and Track B (nonlinear-dominant). All RMSE and MAE values are in units of g; NRMSE is expressed as a percentage.

Method	Track A–Noise-Dominant	Track B–Nonlinear-Dominant
Kalman	$R^{2}=0.234\pm 0.021$ RMSE =0.288±0.006 g NRMSE =14.38±0.31% MAE =0.206±0.004 g	$R^{2}=-24.15\pm 18.76$ RMSE =1.538±0.572 g NRMSE =76.91±28.61% MAE =0.335±0.029 g
ARX (FIR)	$R^{2}=0.226\pm 0.010$ RMSE =0.289±0.004 g NRMSE =14.46±0.20% MAE =0.220±0.004 g	$R^{2}=0.374\pm 0.008$ RMSE =0.260±0.004 g NRMSE =13.00±0.18% MAE =0.191±0.005 g
LPF	$R^{2}=0.687\pm 0.009$ RMSE =0.184±0.004 g NRMSE =9.19±0.18% MAE =0.138±0.004 g	$R^{2}=0.806\pm 0.010$ RMSE =0.145±0.005 g NRMSE =7.24±0.22% MAE =0.096±0.003 g
MLP	$R^{2}=0.832\pm 0.007$ RMSE =0.135±0.003 g NRMSE =6.74±0.12% MAE =0.097±0.003 g	$R^{2}=0.962\pm 0.002$ RMSE =0.064±0.002 g NRMSE =3.19±0.09% MAE =0.042±0.002 g
CNN	$R^{2}=0.926\pm 0.002$ RMSE =0.089±0.001 g NRMSE =4.46±0.05% MAE =0.067±0.001 g	$R^{2}=0.996\pm 0.0003$ RMSE =0.021±0.001 g NRMSE =1.05±0.04% MAE =0.016±0.001 g
LSTM	$R^{2}=0.900\pm 0.003$ RMSE =0.104±0.002 g NRMSE =5.21±0.09% MAE =0.082±0.002 g	$R^{2}=0.993\pm 0.0004$ RMSE =0.027±0.001 g NRMSE =1.35±0.03% MAE =0.020±0.0004 g
Hybrid LPF-CNN	$R^{2}=0.9407\pm 0.0032$ RMSE =0.0800±0.0020 g NRMSE =4.00±0.10% MAE =0.0587±0.0019 g	$R^{2}=0.9970\pm 0.0001$ RMSE =0.0179±0.0002 g NRMSE =0.89±0.01% MAE =0.0132±0.0002 g

## Data Availability

All code and data used in this study are publicly available at https://github.com/faizud/hybrid_lpf_cnn_vibration (version v1.0.0) (accessed on 19 August 2026).

## Abbreviations

The following abbreviations are used in this manuscript:

ARX	AutoRegressive with eXogenous input
CNN	Convolutional Neural Network
EKF	Extended Kalman Filter
FIR	Finite Impulse Response
GELU	Gaussian Error Linear Unit
IMU	Inertial Measurement Unit
LPF	Low-Pass Filter
LSTM	Long Short-Term Memory
MAE	Mean Absolute Error
MEMS	Micro-Electro-Mechanical Systems
MLP	Multi-Layer Perceptron
MSE	Mean Squared Error
NRMSE	Normalized Root Mean Squared Error
PINN	Physics-Informed Neural Network
PSD	Power Spectral Density
RMSE	Root Mean Squared Error
SHM	Structural Health Monitoring
